# Metabolomics Tools for Describing Complex Pesticide Exposure in Pregnant Women in Brittany (France)

**DOI:** 10.1371/journal.pone.0064433

**Published:** 2013-05-21

**Authors:** Nathalie Bonvallot, Marie Tremblay-Franco, Cécile Chevrier, Cécile Canlet, Charline Warembourg, Jean-Pierre Cravedi, Sylvaine Cordier

**Affiliations:** 1 National Institute of Health and Medical Research (INSERM) UMR 1085 IRSET (Research Institute for Environmental and Occupational Health), Rennes, France; 2 EHESP-School of Public Health Rennes, Sorbonne Paris Cité, Paris, France; 3 National Institute for Agricultural Research (INRA) UMR 1331 Toxalim (Research Center in Food Toxicology), University of Toulouse, INP, ENVT, EIP, UPS, UMR1331, Toulouse, France; 4 INSERM UMR 1085 IRSET, Rennes, France; 5 University of Rennes I, Rennes, France; Concordia University Wisconsin, United States of America

## Abstract

**Background:**

The use of pesticides and the related environmental contaminations can lead to human exposure to various molecules. In early-life, such exposures could be responsible for adverse developmental effects. However, human health risks associated with exposure to complex mixtures are currently under-explored.

**Objective:**

This project aims at answering the following questions: What is the influence of exposures to multiple pesticides on the metabolome? What mechanistic pathways could be involved in the metabolic changes observed?

**Methods:**

Based on the PELAGIE cohort (Brittany, France), 83 pregnant women who provided a urine sample in early pregnancy, were classified in 3 groups according to the surface of land dedicated to agricultural cereal activities in their town of residence. Nuclear magnetic resonance-based metabolomics analyses were performed on urine samples. Partial Least Squares Regression-Discriminant Analysis (PLS-DA) and polytomous regressions were used to separate the urinary metabolic profiles from the 3 exposure groups after adjusting for potential confounders.

**Results:**

The 3 groups of exposure were correctly separated with a PLS-DA model after implementing an orthogonal signal correction with pareto standardizations (R2 = 90.7% and Q2 = 0.53). After adjusting for maternal age, parity, body mass index and smoking habits, the most statistically significant changes were observed for glycine, threonine, lactate and glycerophosphocholine (upward trend), and for citrate (downward trend).

**Conclusion:**

This work suggests that an exposure to complex pesticide mixtures induces modifications of metabolic fingerprints. It can be hypothesized from identified discriminating metabolites that the pesticide mixtures could increase oxidative stress and disturb energy metabolism.

## Introduction

The use of pesticides can lead to environmental contaminations to various molecules in different environmental media. Several studies have shown that the proximity to agricultural pesticide applications may be a source of pesticide exposure in addition to domestic or dietary sources [1], [2], [3], [4], [5].

In Brittany, more than 60% of the surface area is devoted to agricultural activities, with a large part (50%) of cereal and corn crops. In this region in the 2000's, almost all land areas received at least four different treatments in order to control annual grasses (herbicides), fungus or insects proliferation (fungicides and insecticides). The main classes used in 2006 were chloroacetanilides, carbamates, morpholines, triazoles, organophosphorus, pyrethroids [6]. For some of these, the modes of action of poisoning in mammals, included human beings are well known: organophosphorus and carbamate insecticides are able to inhibit the acetylcholinesterase, leading to an overstimulation of postsynaptic cholinergic receptors [7]; pyrethroid insecticides can modify the kinetics of voltage-sensitive sodium channels, inducing a change in the nerve action potential [8]. These effects have been confirmed in the case of chronic occupational exposure with moderate levels [9]. Little is known in the case of environmental and low-doses exposure to complex pesticide mixtures in the general population and especially in fetuses and infants, who are considered particularly susceptible to toxicants because of the development of the organism until the puberty. Using biomonitoring tools to assess exposure, several recent studies have suggested potential impact of low-doses exposure to specific pesticide compounds (such organophosphorus insecticides) during pregnancy, on pregnancy outcomes [10], [11] and behavioral and neuropsychological outcomes [12], [13], [14], or triazine herbicides on pregnancy outcomes [15]. Other studies have examined the impact of more complex mixtures on pregnancy outcomes, in using indicators of agricultural activities: In Brittany (France), Petit et al. showed an association between a small head circumference at birth and living in a municipality where peas were grown in early pregnancy [16]. In Colorado (U.S.A.), Xiang et al. suggested an association between low birth weight and total crop, corn crop and sugar beet crop production, using geographic information system in order to identify the proximity of maternal residence to agricultural areas [17]. Schreinemachers studied the rate of birth abnormalities in several U.S. states by comparing counties with a high percentage of wheat land's areas and those with a lower percentage. She showed significant increases in certain birth malformations (circulatory/respiratory and musculoskeletal/integumentary systems) in high wheat counties [18].

Currently, the identification of biomarkers of exposure, early effect and disease is of particular concern, with the development of new high-throughput technologies such as genomics, proteomics or metabolomics. Metabolomics consists in the study of the nature and quantity of all metabolites produced by an organism (including endogenous molecules involved in the growth and homeostasis of cells as well as by-products of external pollutants). It has come to be widely used in recent years to identify metabolic pathways modified by disease, drugs or toxic exposures, as reviewed by several authors [19], [20], [21]. Unlike genomics or proteomics, metabolomics provides extensive data about the phenotype and can be the last step in understanding the functioning of an organism. In theory, this approach might help to characterize biological disruptions caused by various stimuli and environmental factors and thus it could be an integrative tool to increase our understanding of the mode of action induced by pollutant exposures. The metabolic changes could be observed directly in biological fluids, making also possible the direct identification of biomarkers of complex and low-dose exposure or early effect in human population. However, in non-experimental studies the main challenge is to control the high number of factors affecting the metabolome, in addition to environmental exposures (lifestyle, diet, drugs…). To our knowledge, only two epidemiological studies have been interested in the modification in urinary and blood metabolic profiles associated with human environmental pollutant exposures. The first one concerned 51 male workers exposed to welding fumes in Taiwan. The study has shown an increase of metabolites involved in inflammatory and oxidative tissue injury processes, especially glycine, taurine and betaine [22]. The second one was interested in urinary metabolic profiles in 178 human volunteers living near a source of environmental cadmium pollution [23].

For the first time, the present study performed metabolomics analyses on urine of pregnant women with contrasted exposure to pesticide mixtures in order to identify discriminant metabolites between exposure groups. It is based on 2 hypotheses: the metabolome could be modified after an exposure to a toxicant [21] as well as to a low-dose mixture of toxicants; the change of a metabolic process could lead to a disturbance at the cell-scale, affecting possibly the functioning of the whole organism [24].

## Materials and Methods

### Population and sample collection

The INSERM (French National Institute of Health and Medical Research) ethics committee approved the study procedures. The population selected is issued from the PELAGIE cohort, which includes 3421 pregnant women in Brittany (France) enrolled from general population by gynecologists in early pregnancy between 2002 and 2006. Gynecologists informed them about the nature of the study and asked them to participate, after providing written consent. This consent was accompanied by a letter of information describing the goal of the study, the consortium, data collection procedures, follow-up after birth through mailed questionnaires or health examinations. Reference (N°902076; 31 may 2002) to the approval of the National Commission in charge of Data Protection (CNIL) was also indicated. The right to refuse participation and the fact that this refusal would not have any consequence on the relation with her physician was explicitly mentioned. The objective of the PELAGIE study is to assess the consequences of environmental exposures (solvents, persistent organic pollutants, pesticides…) on the pregnancy, birth outcomes and psychomotor development in infant. A detailed description of this cohort is made elsewhere [15]. At her inclusion during the first trimester of the pregnancy (4^th^ to 15^th^ week), each woman had to return a first morning void urine sample that she collected and transferred into two vials containing nitric acid to avoid bacterial degradation. Samples were mailed to the study laboratory in a pre-stamped package at ambient temperature, with routine delivery taking from 1 to 3 days. Upon receipt, the 10 mL samples were frozen and stored at −20°C. In the same time, data on social and demographic characteristics, diet and lifestyle were retrieved by questionnaire. At birth, medical data on health outcomes were obtained.

**Figure 1:**
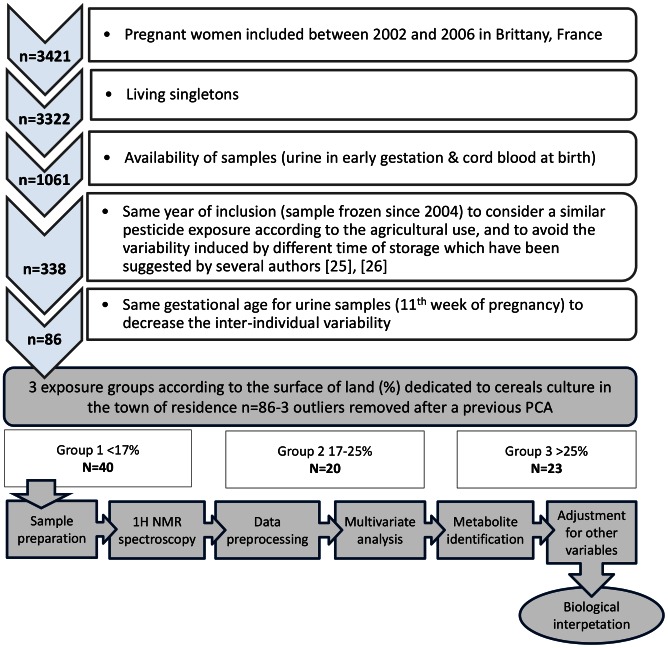
Flowchart of the metabolomics analysis.

For this exploratory study, pregnant women were selected according to 4 criteria as shown in the figure 1. A special attention has been paid to the comparability of the data: the same year of inclusion was selected for all the women to consider a similar likelihood of pesticide exposure according to the agricultural use, and to avoid the potential variability induced by different duration of storage of biological samples [25], [26]. The year 2004 was selected because it corresponds to the higher percentage of inclusion (36%). Finally, only urinary samples from the same gestational age were selected to decrease the variability according to stage of pregnancy suggested in the pilot study (data not shown). Samples were therefore restricted to the 11^th^ week of gestation, corresponding to the highest proportion of subjects (almost 26%, i.e. 86 women).

### Identification of exposure groups

We define groups of exposure according to the surface of land dedicated to agricultural crops in the town of residence of the pregnant woman. Data on agricultural activities were collected from the National General Agricultural Census (RGA) between 2002 and 2006. It provides the percentage of area of the municipality used for agricultural activities according to crop, including corn, wheat, colza, peas, potatoes, and fresh vegetables (plus strawberries and melons). These data were matched with the mother's municipalities of residence in early pregnancy. Because cereal crops are widespread in the study region, we classified the pregnant women in 3 groups according to the percentage of the surface of land dedicated to cereal crops in their town of residence in early pregnancy (First, second and third tercile, corresponding to 0–17%, between >17 and 25% and above 25%). Presence of other crops in the municipalities has been associated with presence of cereals, especially peas crop which has been associated with higher risk of small head circumference according to a previous study [16].

### Metabolomics analyses

#### Sample preparation

After thawing at room temperature and vortexing, 500 µL of urine were mixed with 200 µL of phosphate buffer (pH 7.39) prepared in D_2_O in which was added sodium 3-trimethylsilyl-1-[2,2,3,3,-^2^H_4_]-propionate (TSP, 1 mM). The phosphate buffer is used to minimize variations in chemical shift values in the acquired NMR spectra due to pH differences. TSP served as a chemical shift reference and D_2_O served as a field-frequency lock for the NMR spectrometer. Each sample was vortexed and centrifuged for 10 min at 8,000 rpm to remove any precipitate. Then, 600 µL aliquots were transferred to standard 5 mm - NMR tubes (Norell ST 500, Landisville, NJ) for analysis.

#### Spectra acquisition and metabolite identification

NMR spectra of urine samples were acquired at 300 K on a Bruker Avance DRX-600 operating at 600.13 MHz (Bruker Biospin, Germany) and equipped with an autosampler and an inverse ^1^H-^13^C-^15^N cryoprobe. One-dimensional ^1^H NMR spectra were acquired using a standard pulse sequence NOESY to suppress residual water resonance. A relaxation delay of 2 s and mixing time of 150 ms were used. 128 free induction decays (FIDs) were collected into 32 k data points using a spectral width of 20 ppm with an acquisition time of 3.36 s, giving a total acquisition time of 7.10 s. 2D homonuclear ^1^H-^1^H COSY (correlation spectroscopy) and 2D heteronuclear ^1^H-^13^C HSQC (heteronuclear single quantum coherence spectroscopy) NMR spectra were also acquired for selected samples as an aid to spectral assignment, which was based on matching 1D and 2D data to reference spectra in a home-made reference database, as well as with other databases (http://www.bmrb.wisc.edu/metabolomics/; http://www.hmdb.ca/), and reports in literature.

#### ^1^H NMR data preprocessing

All free induction decays were then multiplied by an exponential function with a line broadening factor of 0.3 Hz prior to Fourier transformation. All spectra were referenced to the chemical shift of TSP (δ 0.00). All NMR spectra were phase- and baseline-corrected manually using Topspin (V2.1, Bruker Biospin, Germany). The spectral region containing residual water and urea resonances (δ 4.515–6.495) was removed and spectra were digitized to 751 buckets corresponding to 0.01 ppm intervals across the chemical shift range δ 0.505–9.995 using the AMIX software package (V3.9.11, Bruker Biospin, Germany). Each integrated region was divided by the total spectral intensity in order to normalize values. This partially removes concentration differences between urine samples.

#### Discriminant analysis on urinary metabolites according to exposure groups

The NMR spectral data were imported into the SIMCA-P+ software package (version 12.0, Umetrics) for multivariate statistical analysis. A preliminary PCA (principal component analysis) was made to remove outliers (n = 3) among the 86 eligible women according to the 4 criteria mentioned above. Additionally, we used orthogonal signal correction (OSC) filtering in order to decrease variability in X-matrix (spectral data) not correlated with the Y-matrix (exposure groups) [27], [28], that is confounding factors such as physiological, experimental, and instrumental factors. Then, filtered data were Pareto-scaled. PLS-DA method was applied to filtered and Pareto scaled data. In PLS-DA, linear combinations of NMR buckets are constructed by maximizing covariance between the Y and X matrices. Then observations are projected onto a few of these linear combinations. Cross-validation was used to determine the number of linear combinations to be included in the PLS-DA model. The quality of the model was given by the two parameters R2Y (proportion of explained variance) and Q2Y (predictive ability). Q2 value was evaluated using a 7-fold cross-validation. A permutation test (200 iterations) was conducted for each PLS-DA model to test for validity. The spectral regions (buckets) with variable importance in the projection (VIP) above 2 were selected in this study. A non-parametric Kruskal-Wallis test with the critical p-value of 0.05 was further used to determine whether a significant difference of each metabolite obtained from PLS-DA models existed between at least two groups. In case of significance, pairwise comparisons were performed (p-value was corrected to take into account multiplicity of comparisons). This test was conducted using the R software (version 2.12.1).

### Adjustments for confounding factors

The previous step was used to identify the metabolites of interest. Polytomous regressions, using spectral data before filtering by OSC, were used to confirm the relations between exposure groups and the concentrations of urinary metabolites previously identified, taking into account individual characteristics of the women. The dependent variable was the exposure group (3 levels) and results from these analyses were reported as the odds ratio (OR) and 95% confidence interval (CI) for each group of exposure (with group 0 as reference) associated with 1 unit increase of the metabolite (treated in continuous). In these analyses, 1 unit = (area under the pic of the bucket of the metabolite/total area of the spectrum) * 10000. The literature suggested some major confounding factors such as age, gender, height or weight, body mass index (BMI) and lifestyle [29], [30], [31], [32]. Educational level (middle/high school, baccalaureate or post-secondary level), maternal age (continuous), parity (1; 2; >2), BMI (continuous), alcohol consumption (no alcohol during pregnancy; occasionally or one glass a day) and smoking status (non-smoker or ex-smoker; stopped smoking in early pregnancy; smoker) were considered as potential confounders and were retained in the model if the likelihood ratio (LR) test was statistically significant for at least one metabolite. Age, parity, BMI and smoking status met this criterion. We used the LR test to select confounders because this one compare nested models in term of goodness of fit. Two missing values were replaced with the median value for BMI and the mode for smoking status. SAS software (version 9.3, SAS Institute) was used for these analyses.

## Results

Table 1 describes the characteristics of enrolled women. Most of them are 25–35 years old (79.5%) with a BMI≤25 (80.7%). The median maternal age at inclusion was 29.3 years old (range 21.5 – 40.9). 59% have a high educational level (post-secondary). 65% already have at least one child and 71.1% didn't smoke while 15.7% stopped during the first trimester. Alcohol consumption was limited with only 13.3% of women reporting an occasional or regular consumption (at least 1 glass a day, only 1 woman). Among the various characteristics studied, only parity differs between the exposure groups (p-value<0.05, see Table 1). Differences were also observed for maternal age, BMI or smoking habits, but these did not attain statistical significance.

**Table 1:** Characteristics of the 83 pregnant women included in the metabolomic study by group of exposure^a^.

	Total	By group of exposure			
		Group 0	Group 1	Group 2	
	(n = 83)	(n = 40)	(n = 20)	(n = 23)	p-value
Characteristics	No. (%)	No. (%)	No. (%)	No. (%)	
**Educational Level**					0.68^b^
Middle/high school	12 (14.5)	8 (20.0)	2 (10.0)	2 (8.7)	
Baccalaureate degree	22 (26.5)	9 (22.5)	7 (35.0)	6 (26.1)	
Post-secondary	49 (59.0)	23 (57.5)	11 (55.0)	15 (65.2)	
**Age**					0.17^b^
<25 years	7 (8.4)	3 (7.5)	3 (15.0)	1 (4.4)	
25–30 years	39 (47.0)	14 (35.0)	9 (45.0)	16 (69.6)	
30–35 years	27 (32.5)	15 (37.5)	7 (35.0)	5 (21.7)	
>35 years	10 (12.0)	8 (20.0)	1 (5.0)	1 (4.4)	
Median [Q1; Q3]	29.3 [27.0; 32.6]	31.5 [26.9; 33.7]	27.8 [26.9; 31.8]	29.1 [27.3; 31.1]	0.15^c^
**Body Mass Index**					0.50^b^
≤25 kg/m^2^	67 (80.7)	30 (76.9)	18 (90.0)	19 (82.6)	
>25 kg/m^2^	15 (19.3)	9 (23.1)	2 (10.0)	4 (17.4)	
*Missing*	*1*	*1*			
Median [Q1; Q3]	21.4 [20.2; 23.8]	21.3 [20.5; 24.7]	21.0 [19.7; 22.2]	21.9 [20.1; 28.3]	0.30^c^
**Parity (including the child to be born)**					0.01^b^
1	29 (34.9)	17 (42.5)	9 (45.0)	3 (13.0)	
2	36 (43.4)	11 (27.5)	9 (45.0)	16 (69.6)	
>2	18 (21.7)	12 (30.0)	2 (10.0)	4 (17.4)	
**Smoking**					0.10^b^
No smoking or ex-smoker	59 (71.1)	30 (76.9)	12 (60.0)	17 (73.9)	
Stop smoking in early pregnancy	13 (15.7)	2 (5.1)	5 (25.0)	5 (21.7)	
Smoking	10 (12.0)	7 (18.0)	3 (15.0)	1 (4.4)	
*Missing*	*1*	*1*			
**Alcohol consumption**					0.84^b^
No alcohol during pregnancy	71 (85.5)	34 (87.2)	18 (90.0)	19 (82.6)	
Occasionally or one glass a day	11 (13.3)	5 (12.8)	2 (10.0)	4 (17.4)	
*Missing*	*1*	*1*			

a Three groups according to the percentage of the surface of land dedicated to cereal crops in the town of residence in early pregnancy: group 0: 0–17%, group 1: >17–25% and group 2: >25%.

b p-value of a Fisher exact test.

c p-value of a Kruskal-Wallis test.

The preliminary PCA identified 3 outliers among the 86 eligible women according to the 4 criteria mentioned in the figure 1. The first woman has a high concentration of urinary glucose and was identified as diabetic. The second one has no specific characteristics compared to the other individuals but a high concentration of hippurate was detected in her urinary sample. A specific dietary habit (benzoate-rich diet for example) may have contributed to this high level of hippurate. The last woman did not appear to have any specific characteristics but her urinary sample was extremely diluted, which could explain a different spectrum compared to the other individuals.

**Figure 2:**
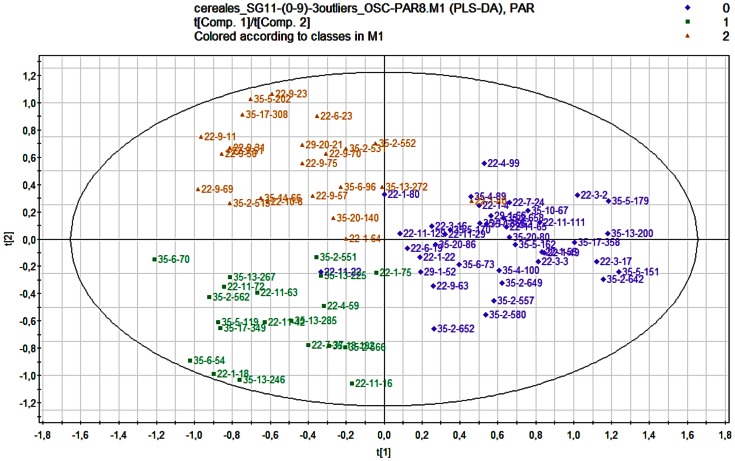
PLS-DA score plot from the ^1^H NMR urinary metabolic profiles from 83 pregnant women. The score plot is the projection of the observations onto the first two latent variables. The PLS-DA model, constructed on OSC-filtered and Pareto-scaled data, includes 4 latent variables (N = 83; R2 = 90.7% and Q2 = 0.53). Three groups according to the percentage of the surface of land dedicated to cereal crops in the town of residence in early pregnancy: purple: group 0: 0–17%, green: group 1: >17–25%; orange: group 2: >25%.

A PLS-DA model comprising four latent variables was constructed on OSC-filtered (eight components removed) and Pareto-scaled data. The 3 exposure groups are correctly discriminated (R2 = 90.7% and Q2 = 0.53). The first two latent variables accounted for a high proportion of total variance (71%). Figure 2 shows projection of the observations (the women) onto the two first principal components (the score plot). This figure shows that the first group (lesser-exposed, called group 0 and corresponding to a 0% to 17% level of surface area of land dedicated to cereal crops in the town of residence) is separated from the more exposed groups (groups 1 and 2) by the first component, while group 1 (cereal >17%–25%) is separated from group 2 (cereal >25%) by the second component.

**Figure 3:**
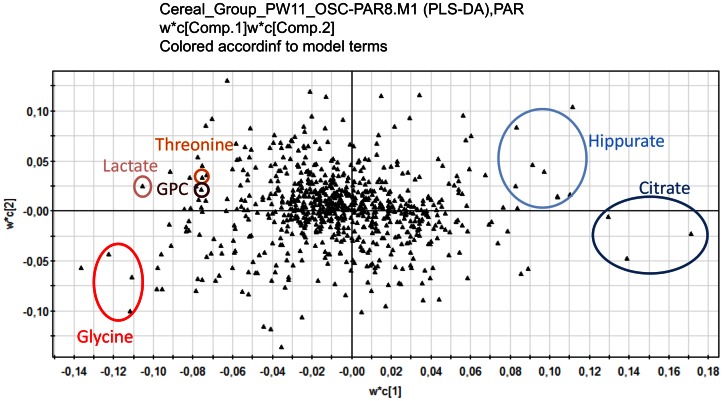
Graphical summary of the correlation between X and Y for the first two components. The correlation between X and Y (w*c) is represented by the loading plot. The PLS-DA model used was constructed on OSC-filtered and Pareto scaled data (N = 83; R2 = 90.7% and Q2 = 0.53), from the ^1^H NMR urinary metabolic profiles from 83 pregnant women differently exposed to pesticides.

More than 60 variables (buckets) were considered important in the PLS-DA model (VIP>2). Figure 3 shows the correlation between variables and components. Among them, 17 variables were statistically significantly different between the first and the other groups of exposure (1 versus 2 and 3 or 1 versus 3) (Kruskal-Wallis test, p<0.05). The most statistically significant changes with OSC data were observed for glycine, lactate, threonine and glycerophosphocholine (GPC) (on the left of the loading plot) with an upward trend, and citrate and hippurate (on the right of the loading plot), with a downward trend. The metabolites associated with their p-values are given in Table 2. Trends in crude associations as estimated by polytomous regressions using spectral data before filtering by OSC (Table 3) were similar to what was observed in table 2 except for hippurate. Adjustment for smoking status, BMI, age and parity, provided trends similar or slightly increased: upward trends for glycine, threonine, lactate and GPC, a downward trend for citrate, and no association for hippurate (Table 3). As an example, compared to exposure group 0 (cereal ≤17%), a unit increase in lactate level multiplies the odds of belonging to exposure group 1 (cereal >17%–25%) and group 2 (cereal >25%) by 1.36 and 1.47 respectively.

**Table 2:** Urinary metabolites discriminated between the 3 groups of pesticide exposures (assessed from the percentage of the surface of land dedicated to cereal crops in the town of residence in early pregnancy) with a PLS-DA model including 4 latent variables on Pareto scaled data (N = 83; R2 = 90.7% and Q2 = 0.53), after an orthogonal signal correction.

Metabolites	Chemical shifts (corresponding to the variables)	Trends	p-values [OSC-data]
**Glycine**	*δ 3,545 ppm*	↗	3.50E-6
**Threonine**	*δ 4,235 ppm*	↗	3.33E-4
**Lactate**	*δ 4,095 ppm*	↗	2.91E-4
**GPC**	*δ 4,285 ppm*	↗	5.00E-4
**Citrate**	*δ 2,665 – 2,545 – 2,525 ppm*	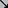	9.72E-6
**Hippurate**	*δ 7,645 – 7,565 – 7,555 – 3,975 ppm*	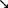	6.39E-5

Abbreviation: GPC: glycerophosphocholine. The trends are observed after an OSC filtering. The significance was assessed with a non-parametric Kruskal-Wallis test (threshold 0.05).

**Table 3:** Association between urinary metabolite changes in pregnant women and exposure to pesticides (assessed from the percentage of the surface of land dedicated to cereal crops in the town of residence in early pregnancy).

Metabolite	Groups of exposure	n	Crude OR (95%CI)	Adjusted^a^ OR (95%CI)	p-value
**Glycine**	0	40	Ref	Ref	
	1	20	1.25 (1.10; 1.43)	1.29 (1.10; 1.52)	0.002
	2	23	1.19 (1.05; 1.35)	1.28 (1.09; 1.50)	0.003
**Threonine**	0	40	Ref	Ref	
	1	20	1.54 (1.04; 2.28)	1.57 (0.99; 2.51)	0.06
	2	23	1.79 (1.21; 2.64)	1.98 (1.21; 3.22)	0.006
**Lactate**	0	40	Ref	Ref	
	1	20	1.35 (1.11; 1.64)	1.36 (1.08; 1.71)	0.008
	2	23	1.38 (1.13; 1.67)	1.47 (1.16; 1.87)	0.002
**GPC**	0	40	Ref	Ref	
	1	20	1.17 (0.98; 1.40)	1.25 (1.00; 1.55)	0.05
	2	23	1.20 (1.01; 1.42)	1.35 (1.07; 1.69)	0.01
**Citrate**	0	40	Ref	Ref	
	1	20	0.98 (0.97; 1.00)	0.98 (0.96; 1.00)	0.03
	2	23	0.98 (0.97 ; 1.00)	0.97 (0.95; 1.00)	0.02
**Hippurate**	0	40	Ref	Ref	
	1	20	1.00 (0.99; 1.01)	0.99 (0.98; 1.00)	0.27
	2	23	1.00 (0.99; 1.01)	1.00 (0.99; 1.01)	0.62

a Adjusted for maternal age, body mass index, parity and smoking status.

## Discussion

**Figure 4:**
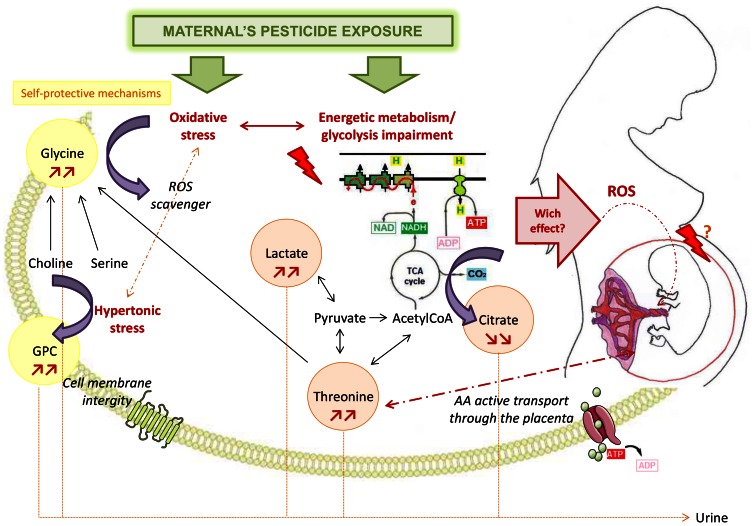
Suggested mechanisms of action of complex and low-dose pesticide mixtures. These suggestions are based on the modification of ^1^H NMR urinary metabolic profile of pregnant women.

This work is an exploratory study designed to test whether the use of metabolomics could help uncover metabolic modifications related to exposure to complex mixtures. It shows an association between the modifications of different urinary metabolites in women in early pregnancy and their exposure to low doses of complex pesticide mixtures. Five metabolites were identified having significantly different urinary concentrations according to the most contrasted exposure groups, after adjusting for maternal age, parity, BMI and smoking habits. Glycine, threonine, lactate and GPC were significantly increased while citrate was decreased. These metabolites are involved in amino-acids metabolism, oxidation/reduction pathways and mitochondrial metabolism (citrate cycle) as shown in Figure 4. Glycine is a cytoprotective agent because it scavenges reactive oxygen species (ROS) and inhibits inflammatory response. It is also a major inhibitory neurotransmitter in the central nervous system [33]. According to previous data showing ROS generation after exposure to organophosphorous or pyrethroids [34], [35], we hypothesize that the increase in urinary glycine could result from a protective mechanism against the oxidative stress induced by a more complex exposure to pesticides. This oxidative stress may induce a mitochondrial dysfunction with an impairment of the tricarboxylic acid (TCA) cycle resulting in a decrease in citrate levels. The observed increase in urinary lactate supports the hypothesis of an alteration of the energy metabolism [36]. GPC plays an important role in the structural integrity of cell membranes [37]. Its increase suggests a protective mechanism against cell damage which could also be a consequence of the oxidative stress. Threonine plays an important role in the TCA cycle. Its catabolism in mammals forms 2-oxobutyrate, glycine and acetylCoA [38]. Its increase could be linked with a disruption in the TCA cycle. But amino-acids such as threonine join the fetal blood through active transport systems in the placenta [39]. An increase in urinary threonine could also be due to an enhancement in plasmatic threonine induced by a disruption of fetoplacental transfers as has been observed in mothers delivering very low birth weight infants [40]. This metabolic change may be a consequence of an adverse effect on the placenta induced by the oxidative stress.

Pesticide exposure was assessed in an indirect way, according to the percentage of cultures in women's towns of residence. There are some limitations to understanding of the relationships between residential proximity to agricultural activities and real individual exposures. However, different studies showed that residential proximity to agricultural pesticide applications could increase exposure to pesticides. Gunier et al. showed a correlation between concentrations of chlorpyrifos, chlorthal-dimethyl, iprodione, phosmet and simazine in house dust (89 dwellings) and the proximity of agricultural activities (within 1,250 m). These concentrations were lower in dwellings without nearby agricultural use [4]. Bradman et al. studied the determinants of organophosphorous exposure in 400 Californian children and showed that among the 12-month-old infants, the concentrations of dialkylphosphate metabolites (coming from organophosphorus) in urine were higher when children lived within 60 m of an agricultural field [5]. Rural residence and close proximity to a farm were also found to be risk factors for pesticide exposure in 190 Chilean children [41]. Therefore, the use of the percentage of cereal cultures in the women's town of residence appears to be a good surrogate for the assessment of complex pesticide exposures. Moreover, preliminary results of an ongoing study on several women of the PELAGIE cohort show that the presence of urinary metabolites of fungicides used in cereal cultures increases with the percentage of these cultures in the town of residence.

Identification of metabolic changes directly in humans may be difficult due to the high number of factors influencing urinary metabolome such as genetics, sex, age, diurnal variation, cultural trends, diet, lifestyle, stress… [42]). Only a limited number of confounding factors were taken into account (age, parity, BMI and smoking status). We noticed that adjustment for these factors did not attenuate the initial trends observed. The PELAGIE population is relatively homogeneous in terms of cultural, dietary trends (women from western France) and age (childbearing age). Regarding diet prior to urine collection and time of collection, we can assume that these uncontrolled factors should be evenly distributed according to exposure group.

In conclusion, this study is a first exploratory work studying the link between metabolic changes and low-dose/complex exposures in environmental health. It can be hypothesized from identified discriminating metabolites that environmental exposure to pesticides could increase oxidative stress and disturb energy metabolism, possibly resulting in disruptions to transplacental exchanges. These observations could have an impact on the offspring but new studies on metabolic profile in newborns are needed to confirm this first hypothesis. Furthermore, an experimental confirmation of oxidative stress hypothesis could be helpful, and this is planned as the next step of this investigation.
